# Oxidative Balance Score Calculated Using Different Methods and Its Associations with Colorectal Cancer Risk

**DOI:** 10.3390/nu17040679

**Published:** 2025-02-14

**Authors:** Fangting Lin, Ruolin Zhou, Qingjian Ou, Kexin Tu, Yujing Fang, Caixia Zhang

**Affiliations:** 1 Department of Epidemiology, School of Public Health, Sun Yat-sen University, Guangzhou 510080, China; linft@mail2.sysu.edu.cn (F.L.); zhourlin@mail2.sysu.edu.cn (R.Z.); tukx@mail2.sysu.edu.cn (K.T.); 2 State Key Laboratory of Oncology in South China, Guangdong Provincial Clinical Research Center for Cancer, Sun Yat-sen University Cancer Center, Guangzhou 510060, China; ouqj@sysucc.org.cn

**Keywords:** oxidative balance score, colorectal cancer, oxidative stress, case-control study

## Abstract

Background: The oxidative balance score (OBS) measures oxidative stress from diet and lifestyle, but research linking it to colorectal cancer (CRC) risk is scarce and varies in calculation methods. Methods: We conducted a case-control study in Guangzhou, China, involving 2799 CRC cases and an equal number of sex- and age-matched controls. We adopted and compared four different methods for calculating the OBSs. The odds ratio (OR) and 95% confidence interval (95%CI) for the relationship between OBS and CRC risk were determined using an unconditional logistic regression model. Restricted cubic splines were used to explore potential non-linear relationships. Additionally, stratified analyses were performed by sex, and subgroup analyses were performed based on the tumor site. Results: Among the four OBSs assessed, OBS-1 demonstrated superior performance. Higher adherence to four OBSs was associated with a lower risk of CRC. The adjusted ORs (95%CIs) for the highest quartile compared to the lowest quartile were as follows: 0.42 (0.35, 0.50) for OBS-1, 0.43 (0.36, 0.51) for OBS-2, 0.50 (0.42, 0.59) for OBS-3, and 0.43 (0.36, 0.51) for OBS-4. Linear relationships were observed between four OBSs and CRC risk (all *p*-Nonlinear > 0.05). Stratified analysis by sex revealed that all four OBSs were negatively associated with CRC risk in both male and female patients. Subgroup analysis by cancer site indicated that four OBSs were negatively associated with the risk of both colon and rectal cancer. Conclusions: All four OBSs were negatively associated with CRC risk, with OBS-1 showing the strongest association in our analysis.

## 1. Introduction

According to the latest estimates from the International Agency for Research on Cancer, there will be 1,926,118 new cases of colorectal cancer (CRC) globally in 2022, making it the third most common cancer worldwide [1]. In China, approximately 592,232 new cases are expected [2]. Research has demonstrated that various factors, including diet, obesity, physical inactivity, and smoking, can influence the risk of developing CRC [3].

Oxidative stress arises when there is an imbalance between reactive oxygen species (ROS) and antioxidants, leading to an excess of ROS, which disrupts redox signaling, impairs molecular control, and causes damage at the cellular level [4]. Studies have shown that oxidative stress and the resulting oxidative damage play a critical role in cancer initiation and progression [5]. Environmental, lifestyle, and dietary factors can all lead to oxidative stress, resulting in elevated ROS levels that may cause DNA damage, mutations, and chromosome instability, ultimately promoting cancer development [6]. Additionally, mast cells and microRNAs have emerged as potential diagnostic and prognostic biomarkers for CRC [7]. Mast cells regulate inflammation and angiogenesis in the tumor microenvironment by releasing cytokines, growth factors, and proteases, often accompanied by ROS production [4,7]. Meanwhile, microRNAs regulate gene expression in important cellular processes [8], and their expression levels can be altered by oxidative stress [9]. Antioxidants such as polyphenols, tocopherols, carotenoids, curcumin, and vitamin C can help mitigate the genotoxic effects of ROS, potentially reducing cancer risk, and antioxidants in food have been associated with positive health outcomes in CRC [10]. Dietary patterns, along with other lifestyle factors, provide a more comprehensive understanding of dietary consumption and may better reflect the exposure to cancer risk compared to any individual food or nutrient [11].

The oxidative balance score (OBS) is a tool used to evaluate an individual’s overall oxidative-reduction status by considering a combination of dietary and lifestyle factors [12]. However, there is no standardized method for calculating OBS, leading to variations in the components used across studies. Dietary antioxidant components commonly include phytochemicals such as α-carotene, β-carotene, β-cryptoxanthin, lycopene, lutein and zeaxanthin, flavonoids, glucosinolates, and vitamins like vitamin C, vitamin E, riboflavin, niacin, vitamin B_6_, folate, and vitamin B_12_. Minerals such as selenium, zinc, copper, calcium, and magnesium, along with dietary fats like n-3 polyunsaturated fatty acids (PUFAs) and dietary fiber, are also frequently included. In contrast, dietary pro-oxidant components often consist of minerals such as iron and various types of fats, including total fats, saturated fatty acids (SFAs), PUFAs, and n-6 PUFAs. Lifestyle factors in the OBS include antioxidant-related components like physical activity and the use of aspirin or other non-steroidal anti-inflammatory drugs (NSAIDs), while pro-oxidant components include smoking, alcohol consumption, and obesity. Most OBSs incorporate over 10 components, with some focusing solely on dietary factors, while others include a broader range of components with varying scoring methodologies [12]. This inconsistency makes it difficult to compare the effects of different OBSs on health outcomes.

To date, the association between the OBS and CRC risk has been examined in two case-control studies [13,14] and four prospective cohort studies [15,16,17,18]. Slattery et al. [13] utilized an OBS that included 11 dietary factors and 2 lifestyle factors, while Bentyaghoob et al. [14] applied an OBS with 10 dietary and 3 lifestyle factors. Dash et al. [15] developed an OBS containing 12 dietary and 4 lifestyle factors, while Mao et al. [16] constructed an OBS incorporating 11 dietary and 4 lifestyle components. Similarly, Chang et al. [17] created an OBS consisting of 17 dietary factors and 5 lifestyle factors. All of these studies concluded that a diet and lifestyle rich in antioxidants was associated with a reduced risk of CRC [13,14,15,16,17]. However, the OBS developed by Gu et al. [18], which included 11 dietary and 3 lifestyle components, found that a higher OBS was associated with a lower CRC risk in women, but not in men.

Existing studies on the relationship between the OBS and CRC risk are limited, with most research focusing on populations in the USA [13,15,16,18], Iran [14], and the UK [17]. Additionally, the OBS methodologies used in these studies vary. To address this, we applied the three most commonly used OBS calculation methods, as proposed by Goodman et al. [19], Dash et al. [20], and Zhang et al. [21], to construct four OBSs to investigate the association between four different OBSs and CRC risk in the Guangdong population of China. We also compared the four OBSs and their individual components. This study examined the association of oxidative stress from dietary and lifestyle factors with the risk of CRC, providing a scientific basis for CRC prevention through modifications in diet and lifestyle. 

## 2. Materials and Methods

### 2.1. Participants

This ongoing case-control study began in July 2010. The cases consisted of newly admitted patients diagnosed with primary colorectal cancer by histopathology within the past three months at the Sun Yat-sen University Cancer Center. Eligible participants were aged 30–75 years and were registered in Guangdong Province or had been long-term residents for at least five years. Patients were excluded if they had a diagnosis of other malignancies, familial adenomatous polyposis, or hereditary non-polyposis CRC, or faced communication barriers such as language issues. Between July 2010 and April 2021, 2985 cases were identified, and 2833 eligible participants were interviewed, with a participation rate of 94.91%. In addition, 34 cases were excluded due to extreme energy intake values (<800 or >4200 kcal d^−1^ for men and <600 or >3500 kcal d^−1^ for women) [22]. Ultimately, 2799 cases participants were included in the study.

Controls were selected using frequency-matching based on age (in five-year intervals) and sex to align with the cases, with participants drawn from the First Affiliated Hospital of Sun Yat-sen University, the Ophthalmology Center of Sun Yat-sen University, and community residents in Guangzhou and Shenzhen. The inclusion and exclusion criteria for controls were similar to those used for cases, with the added requirement that controls could not have any type of cancer. In total, the study included 2799 control participants.

This study adhered to the guidelines established by the Declaration of Helsinki. All study procedures were approved by the Ethical Committee of the School of Public Health at Sun Yat-sen University (approval number 2019-018). Each participant provided informed consent by signing a consent form at the beginning of the interview.

### 2.2. Data Collection

Participants were interviewed face-to-face by trained investigators using the Lifestyle and Dietary Habits and Population Health Questionnaire. This survey gathered socio-demographic characteristics such as age, sex, occupation, marital status, and monthly household income, along with information on dietary habits, lifestyle factors, and other potential confounding factors. Missing data were imputed with the median of the controls or cases according to gender.

Dietary intake was assessed using a food frequency questionnaire (FFQ) that has been evaluated for both reliability and validity [23]. For the case group, the FFQ evaluated dietary intake from the year prior to diagnosis, while for the control group, it assessed dietary intake from the year before the survey. The FFQ covered 7 major food categories, including 12 types of grains, 7 legumes and soy products, 18 vegetables, 28 animal-based foods, 11 fruits, 2 categories of fungi and nuts, and 3 types of beverages and soups, totaling 81 food items. For each food item, participants reported the frequency of consumption (never, daily, weekly, monthly, or annually) and portion sizes, allowing the calculation of daily intake per food item.

Lifestyle habits include details regarding smoking, alcohol consumption, physical activity. Body mass index (BMI) was calculated by dividing an individual’s body weight in kilograms by the square of their height in meters. Smoking history included three categories: current smokers, former smokers, and non-smokers. Information on passive smoking was collected by determining whether non-smokers were exposed to secondhand smoke for more than 15 min on at least one day per week. A regular drinker was defined as someone who consumed alcohol at least once a week for six months or longer, and data on the average daily alcohol intake over the past year were collected. The physical activity assessment collected household and leisure-time activities, as well as occupational activity. Household and leisure-time activities were quantified in metabolic equivalent (MET), while occupational activity was classified into five categories: non-working, sedentary, light, moderate, and heavy. In addition, information on other potential confounders, such as having a first-degree relative with cancer and female menopausal status, was also collected.

Additionally, one case had missing data, resulting in a total of 2798 CRC participants who had blood samples collected early in the morning of the second day after admission, with C-reactive protein (CRP) values obtained through blood testing.

### 2.3. Calculation of the Oxidative Balance Score

In this study, four OBSs were constructed, with higher scores indicating greater antioxidant exposure in the body. Details of the components and assignment methods are provided in Table 1. The daily intakes related to dietary composition for the four OBSs included energy and various nutrients, including vitamin C, vitamin E, zinc, selenium, calcium, magnesium, copper, iron, and fatty acids, based on the China Food Composition 2002 as a reference [24]. Daily intakes of α-carotene, β-carotene, β-cryptoxanthin, lycopene, and lutein and zeaxanthin were determined using the USDA National Nutrition Standards Reference Database [25]. Furthermore, daily intakes of anthocyanins, flavan-3-ols, flavonoids, flavanones, flavanols, isoflavones, and proanthocyanidins were calculated using relevant databases from the United States Department of Agriculture [26,27,28]. Total flavonoid intake was derived from the cumulative sum of these seven subclasses, while daily glucosinolates intake was estimated based on a previous study [29]. Food and nutrient intake data were adjusted using the energy adjustment residual method [30].

OBS-1 was constructed using the calculation method developed by Goodman et al. [19]. It included dietary antioxidant components such as total carotenoids, vitamin C, vitamin E, and selenium, while the dietary pro-oxidant components comprised iron and PUFAs. Additionally, lifestyle factors considered were smoking and alcohol consumption. OBS-1 scores range from 0 to 24.

OBS-2 was created following the methodology proposed by Dash et al. [20]. Its dietary antioxidant components encompassed total carotenoids, flavonoids, glucosinolates, vitamin C, vitamin E, selenium, and n-3 PUFAs. In contrast, the dietary pro-oxidant components included iron, SFAs, and n-6 PUFAs. Lifestyle factors such as smoking, alcohol consumption, obesity, and physical activity were also included. OBS-2 has a scoring range from 0 to 32.

OBS-3 was created based on the method established by Zhang et al. [21]. The dietary antioxidant components included carotene, vitamin C, vitamin E, riboflavin, niacin, vitamin B_6_, total folate, vitamin B_12_, selenium, zinc, copper, calcium, magnesium, and dietary fiber, while the dietary pro-oxidant components consisted of iron and total fat. Lifestyle actors such as cotinine levels, alcohol consumption, BMI, and physical activity were also considered. OBS-3 has a scoring range of 0 to 40.

OBS-4 was constructed by integrating elements from the three previously mentioned OBSs, considering the relationships between each component and oxidative stress. In the context of phytochemicals, the five carotenoids—α-carotene, β-carotene, β-cryptoxanthin, lycopene, and lutein and zeaxanthin—were collectively considered as total carotenoids due to their structural and functional similarities [31]. Flavonoids [32] and glucosinolates [33] were included as antioxidant components. Among vitamins, vitamin C [34] and vitamin E [35] were also classified as antioxidants. In terms of minerals, zinc [36] and selenium [37] were selected for their antioxidant properties, while iron [38] was designated as a pro-oxidant component. For fats, n-3 PUFAs served as an antioxidant component, whereas n-6 PUFAs acted as pro-oxidant factor due to their distinct anti-inflammatory and antioxidant effects [39,40]. SFAs were incorporated as a pro-oxidant component [41]. Lifestyle factors such as smoking [42], alcohol consumption [43], obesity [44], and physical activity [45] were also included. OBS-4 has a scoring range of 0 to 30.

### 2.4. Statistical Analysis

For categorical variables, frequencies and percentages were used for descriptive statistics, and the chi-square test was used to compare differences between cancer cases and controls. For continuous variables, the median (M) and interquartile range (*P*_25_, *P*_75_) were used to describe them, with differences between groups assessed using the Wilcoxon rank-sum test.

Logistic regression models were used to analyze the relationship between different OBSs and CRP. The four OBSs were compared from three aspects: internal consistency, model discrimination, and goodness of fit. Internal consistency was evaluated using Cronbach’s α where higher values indicated better consistency. The discrimination ability of the logistic regression model was assessed using the receiver operating characteristic (ROC) curve and the area under curve (AUC). Higher values indicated better discriminative ability. The goodness of fit and the complexity of the model was evaluated using the Akaike information criterion (AIC). Smaller AIC values indicate an improved fit.

Participants were divided into four quartiles according to different OBSs distribution of male and female controls, with the lowest quartile (Q1) serving as a reference group. The association between different OBSs and CRC risk was analyzed using an unconditional logistic regression model, adjusting for potential confounders that were statistically significant in the basic characteristic of CRC cases and controls and could influence CRC risk. The results were expressed as odds ratios (ORs) with 95% confidence intervals (CIs). A trend test was performed by including the different median OBSs for each quartile group as a continuous variable in the regression model. Additionally, restricted cubic spline (RCS) was used to investigate the dose-response relationship between different OBSs and risk of CRC.

The analysis was stratified by sex (male and female). To assess the interaction between sex and different OBSs quartile, a product term for sex and different OBSs quartile was included in the multivariable model, with the interaction assessed through a likelihood ratio test. Subgroup analysis was performed based on CRC incidence site (colon or rectum) to explore potential differences in the relationship between different OBSs and the two cancer types. The statistical significance of heterogeneity by cancer site was evaluated using the Wald test, with the *p*-Heterogeneity derived from cases only. For sensitivity analysis, each component of the different OBSs was removed individually and included as a covariable in the multivariable model to verify the robustness of the results.

Data analyses were conducted using SPSS version 25.0 and R software version 4.4.1. A two-sided *p*-Value < 0.05 was considered statistically significant.

## 3. Results

### 3.1. Study Population Characteristics

Table 2 presents the socio-demographic and clinical characteristics of the study population. Compared to the controls, the cases were more likely to be married, reside in rural areas, work as farmers or in other occupations, have a first-degree relative with cancer, and have a lower educational level and lower BMI. No significant differences were found between the two groups regarding passive smoking and postmenopausal status (*p*-Value > 0.05).

### 3.2. Comparison of Different OBSs

**Figure 1:**
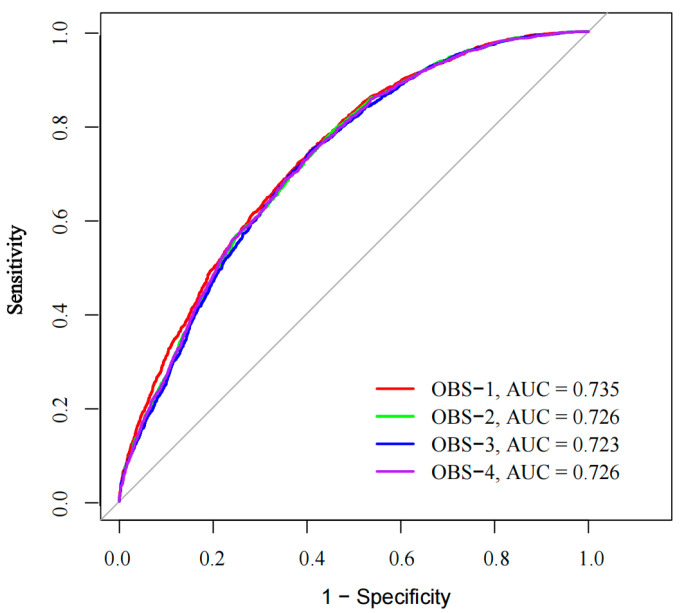
Receiver operating characteristic curves of a multivariable logistic regression model examining the association between oxidative balance score and colorectal cancer risk. OBS, oxidative balance score. The 45° diagonal solid line serves as the reference line, and the red line, the green line, the blue line, and the purple line represent oxidative balance scores 1–4.

A total of 2799 CRC patients were included in the study examining the relationship between the different OBSs and CRP. All four OBSs were inversely associated with increasing levels of CRP, suggesting that these OBSs may have a similar association with CRC risk (all *p*-Trend < 0.001; see Supplementary Materials Table S1). The Cronbach’s α coefficients for the four OBSs, analyzed as continuous and quartile rank variables, were 0.922 and 0.940, respectively, indicating high internal consistency. The ROC curve analysis of the association between different OBSs and CRC risk is detailed in Figure 1. The results showed an AUC value (95%CI) of 0.735 (0.722, 0.747) for OBS-1, 0.726 (0.713, 0.740) for OBS-2, 0.723 (0.709, 0.736) for OBS-3, and 0.726 (0.713, 0.740) for OBS-4, indicating that all four OBSs demonstrate fair discriminative ability. Additionally, the AIC values were 6757.81 for OBS-1, 6825.36 for OBS-2, 6858.31 for OBS-3, and 6832.21 for OBS-4. OBS-1 demonstrated the largest AUC and the lowest AIC, signifying its superior diagnostic performance and goodness of fit.

### 3.3. Association of Different OBSs with CRC Risk

The distribution of the OBS and its components among both cases and controls is shown in Table 3. The scores for all four OBSs were significantly lower in the cases compared to the controls (*p*-Value < 0.001). With the exception of n-3 PUFAs, lutein and zeaxanthin, selenium, and BMI, all other OBS components showed statistically significant differences between the two groups (*p*-Value < 0.05). The median (*P*_25_ and *P*_75_) values of all four OBSs in the cases were lower than those in the controls. The cases exhibited higher daily intakes of SFAs, glucosinolates, niacin, and alcohol compared to the controls, along with a greater preference of smoking.

**Figure 2:**
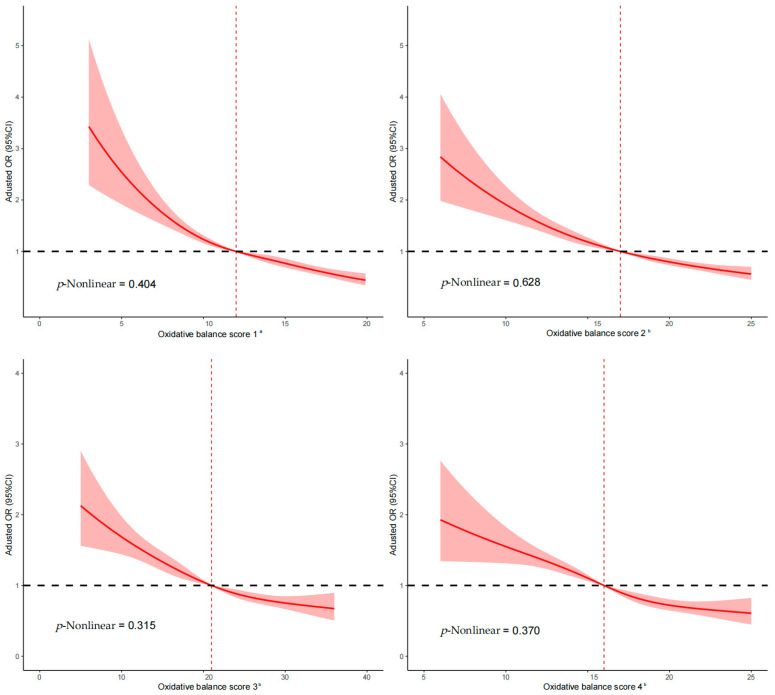
Dose-response relationship between oxidative balance score and colorectal cancer risk. OBS, oxidative balance score; OR, odds ratio; CI, confidence interval. The solid red line indicates the multivariable-adjusted odds ratio, while the light red shaded area represents the 95% confidential intervals. The dashed red line denotes that the median of the first quartile of each score was used as the reference point. ^a^ Adjusted for sex, age, marital status, residence, educational level, occupation, income, first-degree relative with cancer, total energy intake, occupational activity, household and leisure-time activities, and BMI. ^b^ Adjusted for sex, age, marital status, residence, educational level, occupation, income, first-degree relative with cancer, total energy intake, and occupational activity.

As presented in Table 4, all four OBSs were inversely associated with CRC risk (all *p*-Trend < 0.001). The adjusted ORs (95%CIs) for the highest versus the lowest quartile were 0.42 (0.35, 0.50) for OBS-1, 0.43 (0.36, 0.51) for OBS-2, 0.50 (0.42, 0.59) for OBS-3, and 0.43 (0.36, 0.51) for OBS-4. Moreover, the continuous scores for all four OBSs continued to demonstrate an inverse association with CRC risk after multivariable adjustments. The analysis of the continuous relationship between the four OBSs and CRC risk, based on RCS regression models, is illustrated in Figure 2. We discovered L-shaped associations between the four OBSs and CRC risk, indicating that the risk of CRC decreased with increasing scores of the four OBSs, reflecting a linear dose-response relationship (all *p*-Nonlinear > 0.05).

**Figure 3:**
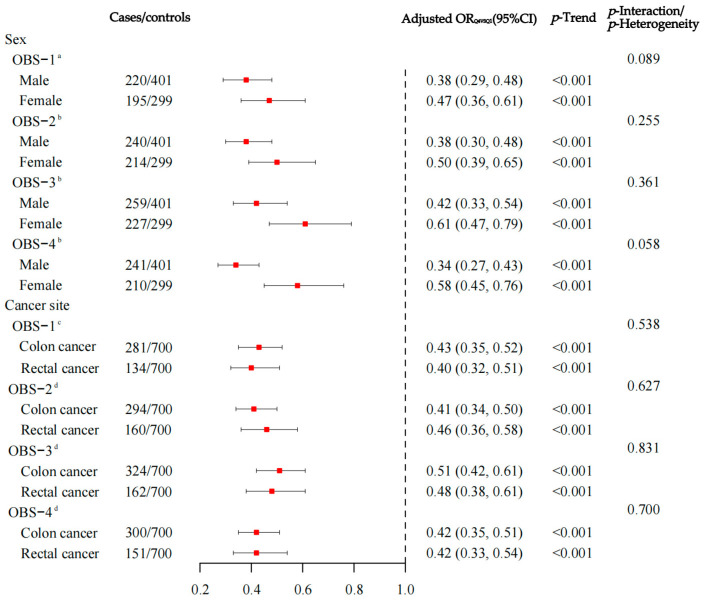
The relationship between oxidative balance score and colorectal cancer risk, with stratified analysis by sex and subgroup analysis by cancer site. OBS, oxidative balance score; Q, quartile; OR, odds ratio; CI, confidence interval. The red boxes indicate the estimate, the length of the black line segment indicates the 95% confidence interval of the estimate, and the black dotted vertical line is used as a reference. ^a^ Adjusted for age, marital status, residence, educational level, occupation, income, first-degree relative with cancer, total energy intake, occupational activity, household and leisure-time activities, and BMI. ^b^ Adjusted for age, marital status, residence, educational level, occupation, income, first-degree relative with cancer, total energy intake, and occupational activity. ^c^ Adjusted for sex, age, marital status, residence, educational level, occupation, income, first-degree relative with cancer, total energy intake, occupational activity, household and leisure-time activities, and BMI. ^d^ Adjusted for sex, age, marital status, residence, educational level, occupation, income, first-degree relative with cancer, total energy intake, and occupational activity.

The sex-stratified analysis revealed that all four OBSs were inversely associated with CRC risk among both men and women (all *p*-Trend < 0.001), aligning with the findings from the general population. The interaction between the four OBSs and sex regarding CRC risk was non-significant (all *p*-Interaction > 0.05). Among the cases, 1794 individuals were diagnosed with colon cancer, while 1005 were diagnosed with rectal cancer. Subgroup analyses showed consistent results with the overall CRC findings, demonstrating a significant association between the four OBSs and the risks of both colon and rectal cancer (all *p*-Trend < 0.001). The heterogeneity tests among the four OBSs concerning colon and rectal cancer were non-significant (*p*-Heterogeneity > 0.05) (Figure 3). Additionally, sensitivity analyses confirmed that the significant associations between the four OBSs and CRC risk remained consistent (see Supplementary Materials Table S2).

## 4. Discussion

This large case-control study aimed to examine the association between four OBSs and CRC risk in the Chinese population. The findings revealed that all four OBSs were inversely associated with CRP and demonstrated an inverse association with CRC risk in the general population. These results were consistent across analyses results conducted by sex and cancer site.

Consistent with previous studies, our study found an inverse association between the OBS and the risk of CRC. Slattery et al. [13] conducted a case-control study in the USA, which included 1555 colon cancer cases and 1956 controls, along with 754 rectal cancer cases and 959 controls. They reported that the OBS was linked to a reduced risk of both colon and rectal cancer, with adjusted OR for the fourth quartile compared to the first quartile of 0.52 (95%CI: 0.41, 0.66) for colon cancer and 0.49 (0.35, 0.70) for rectal cancer. The OBS used in their study was similar to OBS-1, but it additionally included vitamin D, total folate, and calcium, while excluding α-carotene, β-cryptoxanthin, and alcohol consumption. In a small case-control study involving 71 Iranian cases and 142 controls, Bentyaghoob et al. [14] found that the OBS was associated with a reduced risk of CRC, with an adjusted OR for the third tertile compared to the first tertile of 0.23 (95%CI: 0.07, 0.72). Their OBS was based on OBS-1, with the addition of obesity, SFAs, vitamin B_9_, and zinc, while removing alcohol consumption, α-carotene, β-cryptoxanthin, lycopene, and lutein and zeaxanthin. Dash et al. [15] observed from the Cancer Prevention Study II (CPS-II) Nutrition Cohort that their OBS, which aligned with the components of OBS-2, was associated with a reduced risk of CRC, with an adjusted relative risk for the fourth quartile compared to the first quartile of 0.59 (95%CI: 0.49, 0.70). Similarly, Mao et al. [16] found that participants in the prospective Iowa Women’s Health Study (IWHS) demonstrated an inverse relationship between OBS and CRC risk, showing an adjusted hazard ratio for the fifth quintile compared to the first quintile of 0.66 (95%CI: 0.54, 0.80). The OBS utilized in their study was largely similar to OBS-2, although it excluded β-cryptoxanthin among the total carotenoids and omitted the glucosinolates component due to lack of data. Chang et al. [17] also found that higher antioxidant exposure, as assessed by the OBS, was associated with a lower CRC risk among UK Biobank participants. Their OBS was largely based on OBS-2 but incorporated additional components such as SFAs, PUFAs, meat, retinol, vitamin D, tea, and vegetables, while removing certain minerals, including niacin, selenium, zinc, copper, and calcium.

Oxidative stress is a critical factor in colorectal carcinogenesis [46]. Variations in the generation of ROS can influence the levels of oxidized proteins, lipids, and DNA damage, thus increasing susceptibility to colon cancer [47]. Antioxidants have the ability to donate electrons, neutralizing free radicals and preventing cellular damage; thus, natural and dietary antioxidants may possess inherent anticancer properties [48]. Research has shown that serum albumin and red cell distribution width partially mediated the associations between dietary OBS and gastrointestinal cancers [49]. Another study reported that serum albumin, uric acid, and neutrophil count significantly mediate the association between the OBS and CRC [17], suggesting that dietary factors may influence cancer risk through their impact on these biomarkers. Additionally, dietary carotenoids enhance the body’s antioxidant defense system by interacting with transcription factors such as nuclear factor erythroid 2-related factor 2 (Nrf-2), as well as by acting as quenchers of ROS [50]. Flavonoids can modulate the inflammatory response and oxidative stress associated with tumors through various anti-inflammatory mechanisms, including the nuclear factor kappaB (NF-κB) pathway, which plays a crucial role in the progression from inflammation to colon cancer [51]. Selenium-based antioxidants protect against metal-mediated DNA damage through metal coordination [37]. Fish oil, rich in n-3 PUFAs, helps defend against the activation of peroxisome proliferator-activated receptor-alpha (PPARα) and promotes the increased expression of antioxidant genes, thereby providing protection against excess ROS [52]. The antioxidant capacity of vitamin C is demonstrated by its ability to rapidly reduce superoxide ion (O_2_−) and singlet oxygen through dehydrogenation [10]. Vitamin E scavenges peroxyl radicals, breaking the chain of propagation, and its synergistic effect with vitamin C significantly enhances overall antioxidant capacity [53]. Lifestyle factors are also important exogenous contributors to oxidative stress [54]. Physical activity, especially vigorous exercise, generates ROS through skeletal muscle contraction and simultaneously upregulates the muscle antioxidant defense system [55]. Additionally, cigarette smoke [6], ethanol metabolism from alcohol consumption [56], and adipokines associated with obesity [57] are linked to increased free radical production in the body. Therefore, the OBS, which integrates antioxidant and pro-oxidant factors from diet and lifestyle, is biologically associated with the risk of CRC.

Stratified analysis by sex showed that all four OBSs were inversely associated with CRC risk in both men and women. This finding aligns with the results reported by Dash et al. [15] and Chang et al. [17]. However, in the Prostate, Lung, Colorectal, and Ovarian (PLCO) Cancer Screening Trial, an inverse association between the OBS and CRC risk was observed in women, with an adjusted hazard ratio for the fifth quintile compared to the first quintile of 0.72 (95%CI: 0.52, 0.99), but not in men [18]. The OBS used in the PLCO trial included greater emphasis on the use of aspirin and other NSAIDs compared to OBS-1, as well as accounting for supplement intake of β-carotene, vitamin C, vitamin E, selenium, and iron. In addition, men in the PLCO trial exhibited higher BMI, smoking history and intensity, and alcohol consumption and intensity, alongside lower dietary antioxidants intake, which may explain the lack of association between OBS and CRC risk in this group. Currently, there is limited research on sex differences regarding the relationship between the OBS and CRC risk, and further investigation is needed to determine whether the association is modified by sex.

Subgroup analysis based on the cancer site showed that all four OBSs were inversely associated with the risk of both colon and rectal cancer. This finding is consistent with the results of Dash et al. [15], who also reported that the association between the OBS and CRC risk did not vary by tumor location (distal colon, proximal colon, or rectum). In contrast, Mao et al. [16] found no significant association between the OBS and different sites in their subgroup analysis of a cohort of older women. Their study focused solely on women aged 55–69 from the IWHS, which differs significantly from the participant demographics in our study. Moreover, the IWHS subgroups contained a relatively small number of female cases—873 in the proximal colon, 400 in the distal colon, and 312 in the rectum—which may have contributed to the discrepancies in results.

To our knowledge, this study is the first to explore the relationship between the OBS and the risk of CRC in the Chinese population. We aimed to comprehensively include all dietary and lifestyle components of the OBS from previous research. The validity of different OBSs was confirmed using CRP, and we compared different OBSs based on internal consistency, model fit, and discriminative ability. The FFQ used in this study aligns with the dietary patterns of the Guangdong population, has been assessed for reliability and validity, and has been employed in a number of similar studies [58,59], effectively capturing true dietary intake.

Our study had certain limitations. First, the CRC cases were sourced exclusively from one hospital within the Sun Yat-sen University Cancer Center, which may introduce potential selection bias. However, as the largest cancer hospital in Southern China, its cases characteristics align closely with those found in Guangdong Province and surrounding areas [60,61]. Moreover, the controls were drawn from various regions of Guangdong, achieving a high response rate that may help reduce selection bias to some extent. Second, using a FFQ to collect dietary information could lead to recall bias. To reduce this bias, participants were carefully selected according to strict inclusion and exclusion criteria, and only those diagnosed less than three months prior were recruited. Third, social desirability bias and reporting bias may have influenced responses during data collection, particularly regarding alcohol consumption, as participants might have felt stigmatized or pressured to provide more socially acceptable answers. To address this, we assured participants of the survey’s anonymity and confidentiality while not disclosing its specific aims. Fourth, some participants dropped out of the survey due to boredom, potentially skewering the results in favor of those who completed the questionnaire patiently, which may lead to selection bias. To address this, we simplified the questions, placed interesting or easy-to-answer questions first, and informed respondents of the survey’s purpose and the estimated completion time to improve preparation. Fifth, the length of the interview may have caused participants to lose focus. To counteract this, we skipped irrelevant questions based on participants’ responses, reducing unnecessary questions, and included interesting pictures to maintain participants’ interest. Finally, the study did not collect data on the use of aspirin, other NSAIDs, nutrient supplements, or direct markers of oxidative stress. Additionally, all components of the OBS were equally weighted, which may not accurately reflect their true biological contributions. However, previous studies suggest that whether components are weighted or not does not significantly affect the relationship between the OBS and outcomes [15,62].

## 5. Conclusions

This large case-control study showed that diets and lifestyles characterized by greater antioxidant exposure relative to pro-oxidant factors were associated with a lower risk of CRC. Among the four OBSs calculated using different methods, OBS-1—which included total carotenoids, vitamin C, vitamin E, selenium, iron, PUFAs, smoking, and alcohol consumption—demonstrated the strongest association with CRC risk. The findings offer a scientific basis for CRC prevention by promoting dietary and lifestyle modifications and guiding the development of personalized dietary recommendations for different risk groups. Specifically, high-risk individuals may benefit from increased antioxidant intake through diet and lifestyle adjustments, while reducing pro-oxidant dietary and lifestyle factors.

## Figures and Tables

**Table 1:** Oxidative balance score components and assignment methods.

Components	OBS-1	OBS-2	OBS-3	OBS-4	Assignment Mode
Dietary antioxidant components					Assign values of 0, 1, and 2 according to the lowest to highest tertile
Total carotenoids	√	√	—	√	
Carotene	—	—	√	—	
α-carotene	—	—	—	—	
β-carotene	—	—	—	—	
β-cryptoxanthin	—	—	—	—	
Lycopene	—	—	—	—	
Lutein and zeaxanthin	—	—	—	—	
Flavonoids	—	√	—	√	
Glucosinolates	—	√	—	√	
Vitamin C	√	√	√	√	
Vitamin E	√	√	√	√	
Riboflavin	—	—	√	—	
Niacin	—	—	√	—	
Vitamin B_6_	—	—	√	—	
Total folate	—	—	√	—	
Vitamin B_12_	—	—	√	—	
Selenium	√	√	√	√	
Zinc	—	—	√	√	
Copper	—	—	√	—	
Calcium	—	—	√	—	
Magnesium	—	—	√	—	
n-3 PUFAs	—	√	—	√	
Dietary fiber	—	—	√	—	
Dietary pro-oxidant components					Assign values of 2, 1, and 0 according to the lowest to highest tertile
Iron	√	√	√	√	
Total fat	—	—	√	—	
SFAs	—	√	—	√	
PUFAs	√	—	—	—	
n-6 PUFAs	—	√	—	√	
Lifestyle antioxidant components					Assign values of 0, 1, and 2 according to the lowest to highest tertile
Physical activity	—	√	√	√	
Lifestyle pro-oxidant components					
Smoking history	√	√	√	√	Non-smokers were given a value of 2, former smokers were given a value of 1, and current smokers were given a value of 0
Alcohol consumption	√	√	√	√	Never drinking was assigned a value of 2, while drinkers scored 1 above the median daily alcohol consumption and 0 below it
Obesity	—	√	√	√	BMI ≤ 23.9 kg/m^2^ was assigned 2, 24.0–27.9 kg/m^2^ was assigned 1, and ≥28.0 kg/m^2^ was assigned 0
Score	0–24	0–32	0–40	0–30	

OBS, oxidative balance score; PUFAs, polyunsaturated fatty acids; SFAs, saturated fatty acids; BMI, body mass index. A checkmark (√) signifies that the component was included, while a dash (—) indicates that the component was not included. Total carotenoids were the sum of α-carotene, β-carotene, β-cryptoxanthin, lycopene, and lutein and zeaxanthin. Carotene was defined as the sum of α-carotene and β-carotene.

**Table 2:** The basic characteristic of colorectal cancer cases and controls.

Characteristics	Cases (n = 2799)	Controls (n = 2799)	*p*-Value ^a^
Age (years), median (*P*_25_, *P*_75_)	58.47 (49.84, 65.06)	58.33 (50.22, 64.67)	0.741
BMI (kg/m^2^), median (*P*_25_, *P*_75_)	23.23 (20.96, 25.39)	23.50 (21.48, 25.53)	0.004
Men, n (%)	1603 (57.27)	1603 (57.27)	1.000
Married, n (%)	2661 (95.07)	2554 (91.25)	<0.001
Urban residence, n (%)	1802 (64.38)	2169 (77.49)	<0.001
Educational level, n (%)			<0.001
Primary school or below	870 (31.08)	619 (22.12)	
Middle school	785 (28.05)	713 (25.47)	
High school/technical school	681 (24.33)	751 (26.83)	
College or above	463 (16.54)	716 (25.58)	
Occupation, n (%)			0.013
Administrator	395 (14.11)	475 (16.97)	
Blue collar worker	624 (22.29)	608 (21.72)	
Famer/other	1780 (63.59)	1716 (61.31)	
Income, CNY/month, n (%)			<0.001
≤2000	379 (13.54)	358 (12.79)	
2001–5000	935 (33.40)	1085 (38.76)	
5001–8000	830 (29.65)	840 (30.01)	
≥8001	655 (23.40)	516 (18.44)	
Ever smoker, n (%)	1103 (39.41)	859 (30.69)	<0.001
Passive smoker, n (%)	793 (28.33)	802 (28.65)	0.813
Regular drinker, n (%)	504 (18.01)	400 (14.29)	<0.001
Occupational activity, n (%)			<0.001
Non-working	334 (11.93)	968 (34.58)	
Sedentary	798 (28.51)	587 (20.97)	
Light	773 (27.62)	653 (23.33)	
Moderate	417 (14.90)	263 (9.40)	
Heavy	477 (17.04)	328 (11.72)	
First-degree relative with cancer, n (%)	416 (14.86)	238 (8.50)	<0.001
Postmenopausal status ^b^, n (%)	865 (72.32)	891 (74.50)	0.247

BMI, body mass index. ^a^ Continuous variables were assessed by Wilcoxon rank-sum tests. Categorical variables were evaluated by χ^2^ tests. ^b^ Among the woman subgroup.

**Table 3:** Oxidative balance score and its components of colorectal cancer cases and controls.

Characteristics	Cases (n = 2799)	Controls (n = 2799)	*p*-Value ^b^
OBS			
OBS-1 ^a^	12.00 (9.00, 15.00)	13.00 (10.00, 16.00)	<0.001
OBS-2 ^a^	16.00 (13.00, 20.00)	18.00 (15.00, 21.00)	<0.001
OBS-3 ^a^	19.00 (13.00, 26.00)	22.00 (16.00, 28.00)	<0.001
OBS-4 ^a^	16.00 (13.00, 19.00)	17.00 (14.00, 20.00)	<0.001
Dietary OBS components			
Energy (kcal/d) ^a^	1481.09 (1197.73, 1815.78)	1543.13 (1259.02, 1943.86)	<0.001
Total carotenoids (μg/d) ^a,c^	11,710.26 (8068.39, 16,080.51)	12,587.42 (8985.86, 17,068.13)	<0.001
Carotene (μg/d) ^a,c^	5848.17 (4062.97, 8023.67)	6305.53 (4501.68, 8576.75)	<0.001
α-carotene (μg/d) ^a,c^	482.05 (246.61, 848.37)	561.60 (319.63, 999.00)	<0.001
β-carotene (μg/d) ^a,c^	5272.67 (3677.69, 7198.67)	5662.73 (4083.96, 7623.21)	<0.001
β-cryptoxanthin (μg/d) ^a,c^	51.60 (31.29, 80.41)	69.01 (45.05, 104.64)	<0.001
Lycopene (μg/d) ^a,c^	487.09 (248.04, 898.95)	720.45 (390.73, 1175.04)	<0.001
Lutein and zeaxanthin (μg/d) ^a,c^	4852.77 (3060.80, 7378.31)	4950.92 (3330.84, 7184.95)	0.090
Flavonoids (mg/d) ^a, c^	91.28 (61.94, 129.92)	108.98 (76.43, 151.40)	<0.001
Glucosinolates (mg/d) ^a,c^	128.27 (80.19, 192.47)	117.46 (71.02, 174.46)	<0.001
Vitamin C (mg/d) ^a,c^	135.06 (95.07, 183.92)	142.16 (103.16, 187.34)	<0.001
Vitamin E (mg/d) ^a,c^	9.60 (7.54, 12.14)	10.66 (8.42, 13.49)	<0.001
Niacin (mg/d) ^a,c^	15.16 (13.12, 17.53)	15.00 (12.90, 17.09)	0.002
Vitamin B_6_ (mg/d) ^a,c^	0.82 (0.69, 0.95)	0.85 (0.74, 0.99)	<0.001
Folate (μg/d) ^a,c^	208.88 (178.10, 245.94)	223.03 (190.70, 261.71)	<0.001
Vitamin B_12_ (mg/d) ^a,c^	1.73 (1.19, 2.41)	1.86 (1.33, 2.46)	<0.001
Riboflavin (mg/d) ^a,c^	0.84 (0.70, 1.01)	0.90 (0.76, 1.07)	<0.001
Selenium (μg/d) ^a,c^	17.48 (15.32, 19.96)	17.43 (15.40, 19.77)	0.550
Iron (mg/d) ^a,c^	49.65 (40.07, 61.69)	52.50 (43.66, 63.12)	<0.001
Zinc (mg/d) ^a,c^	10.77 (9.54, 12.16)	10.54 (9.30, 11.83)	<0.001
Copper (mg/d) ^a,c^	1.83 (1.53, 2.23)	1.96 (1.62, 2.51)	<0.001
Calcium (mg/d) ^a,c^	371.87 (290.32, 476.79)	421.77 (327.78, 544.42)	<0.001
Magnesium (mg/d) ^a,c^	236.40 (207.27, 268.78)	242.21 (213.43, 274.56)	<0.001
Total fat (g/d) ^a,c^	32.02 (23.74, 43.17)	33.17 (26.31, 41.79)	0.001
SFAs (g/d) ^a,c^	8.78 (6.22, 12.03)	7.95 (5.16, 11.16)	<0.001
PUFAs (g/d) ^a,c^	4.69 (3.74, 5.76)	4.86 (3.90, 6.03)	<0.001
n-6 PUFAs (g/d) ^a,c^	4.12 (3.22, 5.28)	4.50 (3.55, 5.58)	<0.001
n-3 PUFAs (g/d) ^a,c^	0.78 (0.56, 1.05)	0.80 (0.60, 1.03)	0.058
Dietary fiber (g/d) ^a,c^	8.45 (6.91, 10.27)	9.26 (7.68, 11.00)	<0.001
Lifestyle OBS components			
Alcohol consumption (g/d) ^a,d^	99.32 (35.71, 225.00)	26.12 (1.97, 125.00)	<0.001
Household and leisure-time activities (MET-h/week) ^a^	27.56 (8.50, 52.50)	34.50 (16.00, 56.06)	<0.001
Smoking history, n (%)			<0.001
Current	772 (27.58)	588 (21.01)	
Former	373 (13.33)	297 (10.61)	
Non-smoking	1654 (59.09)	1914 (68.38)	
BMI (kg/m^2^), n (%)			0.094
≤23.9	1666 (59.52)	1601 (57.20)	
24.0–27.9	925 (33.05)	1002 (35.80)	
≥28.0	208 (7.43)	196 (7.00)	

OBS, oxidative balance score; PUFAs, polyunsaturated fatty acids; SFAs, saturated fatty acid; BMI, body mass index. Total carotenoids were the sum of α-carotene, β-carotene, β-cryptoxanthin, lycopene, and lutein and zeaxanthin. Carotene was the sum of α-carotene and β-carotene. ^a^ Data are presented as median (*P*_25_, *P*_75_). ^b^ Continuous variables were assessed by Wilcoxon rank-sum tests. Categorical variables were evaluated by χ^2^ tests. ^c^ Adjust food and nutrient intake using energy residual method. ^d^ Drinkers were asked about their alcohol consumption over the past year.

**Table 4:** Logistic regression analysis of the association between oxidative balance score and colorectal cancer risk.

	Q1	Q2	Q3	Q4	*p*-Trend	Continuous (per SD Increment)
OBS-1						
No. of cases/controls	1067/699	781/700	536/700	415/700		
Crude OR (95%CI)	1.00	0.73 (0.64, 0.84)	0.50 (0.43, 0.58)	0.39 (0.33, 0.45)	<0.001	0.90 (0.88, 0.91)
Adjusted OR (95%CI) ^a^	1.00	0.72 (0.62, 0.83)	0.49 (0.42, 0.58)	0.42 (0.35, 0.50)	<0.001	0.90 (0.88, 0.91)
OBS-2						
No. of cases/controls	1011/699	767/700	567/700	454/700		
Crude OR (95%CI)	1.00	0.76 (0.66, 0.87)	0.56 (0.48, 0.65)	0.45 (0.38, 0.52)	<0.001	0.93 (0.91, 0.94)
Adjusted OR (95%CI) ^b^	1.00	0.72 (0.61, 0.83)	0.54 (0.46, 0.64)	0.43 (0.36, 0.51)	<0.001	0.92 (0.91, 0.93)
OBS-3						
No. of cases/controls	947/699	775/700	591/700	486/700		
Crude OR (95%CI)	1.00	0.82 (0.71, 0.94)	0.62 (0.54, 0.72)	0.51 (0.44, 0.60)	<0.001	0.96 (0.96, 0.97)
Adjusted OR (95%CI) ^b^	1.00	0.81(0.70, 0.95)	0.60 (0.51, 0.71)	0.50 (0.42, 0.59)	<0.001	0.96 (0.95, 0.97)
OBS-4						
No. of cases/controls	976/699	740/700	632/700	451/700		
Crude OR (95%CI)	1.00	0.76 (0.66, 0.87)	0.65 (0.56, 0.75)	0.46 (0.40, 0.54)	<0.001	0.94 (0.92, 0.95)
Adjusted OR (95%CI) ^b^	1.00	0.75 (0.64, 0.88)	0.62 (0.53, 0.72)	0.43 (0.36, 0.51)	<0.001	0.93 (0.92, 0.94)

OBS, oxidative balance score; Q, quartile; OR, odds ratio; CI, confidence interval; SD, standard deviation. ^a^ Adjusted for sex, age, marital status, residence, educational level, occupation, income, first-degree relative with cancer, total energy intake, occupational activity, household and leisure-time activities, and BMI. ^b^ Adjusted for sex, age, marital status, residence, educational level, occupation, income, first-degree relative with cancer, total energy intake, and occupational activity.

## Data Availability

The data that support the findings of this study are available from the corresponding author upon reasonable request.

## Supplementary Materials

The following supporting information can be downloaded at: https://www.mdpi.com/article/10.3390/nu17040679/s1, Table S1: The relationship between oxidative balance score and C-reactive protein. Table S2: Sensitivity analysis of the relationship between oxidative balance score and colorectal cancer risk.

## Abbreviations

The following abbreviations are used in this manuscript:

AIC	Akaike information criterion
AUC	Area under curve
CI	Confidence interval
CRC	Colorectal cancer
CRP	C-reactive protein
FFQ	Food frequency questionnaire
MET	Metabolic equivalent
NSAIDs	Non-steroidal anti-inflammatory drugs
OBS	Oxidative balance score
OR	Odds ratio
PUFAs	Polyunsaturated fatty acids
Q	Quartile
ROC	Receiver operating characteristic
ROS	Reactive oxygen species
SFAs	Saturated fatty acids
SD	Standard deviation
