# Supplementary material for: Oxidative Balance Score Calculated Using Different Methods and Its Associations with Colorectal Cancer Risk
Source: Nutrients. 2025 Feb 14;17(4):679. doi: 10.3390/nu17040679 (PMC11858281; doi:10.3390/nu17040679)
Supplement: Supplementary file 1 [file nutrients-17-00679-s001.zip › nutrients-3443117-supplementary.pdf]

Table S1. The relationship between oxidative balance score and C-reactive protein.

	Q1	Q2	Q3	Q4	*p*-Trend
OBS-1					
No. of events / n	230/698	186/700	191/700	154/700	
Adjusted OR (95%CI) [a]	1.00	0.74 (0.59, 0.93)	0.77 (0.61, 0.96)	0.57 (0.45, 0.73)	<0.001
OBS-2					
No. of events / n	229/698	201/700	163/700	168/700	
Adjusted OR (95%CI) [b]	1.00	0.82 (0.65, 1.03)	0.62 (0.49, 0.78)	0.63 (0.50, 0.80)	<0.001
OBS-3					
No. of events / n	209/698	220/700	169/700	163/700	
Adjusted OR (95%CI) [b]	1.00	1.08 (0.86, 1.36)	0.74 (0.59, 0.94)	0.70 (0.55, 0.89)	<0.001
OBS-4					
No. of events / n	226/698	200/700	176/700	159/700	
Adjusted OR (95%CI) [b]	1.00	0.83 (0.66, 1.04)	0.70 (0.55, 0.88)	0.60 (0.47, 0.76)	<0.001

Abbreviations: OBS, oxidative balance score; CRP, C-reactive protein; OR, odds ratio; CI, confidence interval. Q, quartile. [a] Adjusted for age, sex, total energy intake, BMI, and household and leisure-time activities. [b] Adjusted for age, sex, and total energy intake.

Table S2. Sensitivity analysis of the relationship between oxidative balance score and colorectal cancer risk.

	Adjusted OR (95%CI) [a]				
	Q1	Q2	Q3	Q4	*p*-Trend
OBS-1					
Excluded PUFAs	1.00	0.61（0.52，0.71）	0.45（0.38，0.53）	0.40（0.33，0.48）	<0.001
Excluded α-carotene	1.00	0.61（0.53，0.72）	0.53（0.45，0.62）	0.35（0.29，0.42）	<0.001
Excluded β-carotene	1.00	0.62（0.53，0.72）	0.44（0.36，0.52）	0.23（0.19，0.29）	<0.001
Excluded β-cryptoxanthin	1.00	0.70（0.60，0.82）	0.71（0.60，0.84）	0.49（0.40，0.59）	<0.001
Excluded lycopene	1.00	0.65（0.55，0.76）	0.62（0.53，0.73）	0.42（0.35，0.50）	<0.001
Excluded lutein and zeaxanthin	1.00	0.58（0.50，0.68）	0.45（0.38，0.53）	0.27（0.22，0.33）	<0.001
Excluded vitamin C	1.00	0.64（0.54，0.74）	0.41（0.35，0.49）	0.23（0.19，0.29）	<0.001
Excluded vitamin E	1.00	0.65（0.55，0.75）	0.58（0.49，0.68）	0.38（0.31，0.45）	<0.001
Excluded selenium	1.00	0.50（0.42，0.58）	0.62（0.53，0.73）	0.39（0.33，0.47）	<0.001
Excluded iron	1.00	0.67（0.57，0.78）	0.47（0.39，0.56）	0.24（0.20，0.30）	<0.001
Excluded alcohol consumption	1.00	0.68（0.58，0.79）	0.42（0.35，0.49）	0.34（0.28，0.41）	<0.001
Excluded smoking history	1.00	0.78（0.67，0.91）	0.54（0.46，0.63）	0.44（0.37，0.52）	<0.001
OBS-2					
Excluded SFAs	1.00	0.75（0.64，0.87）	0.56（0.48，0.66）	0.49（0.41，0.58）	<0.001
Excluded n-6 PUFAs	1.00	0.69（0.59，0.81）	0.52（0.44，0.62）	0.43（0.37，0.52）	<0.001
Excluded n-3 PUFAs	1.00	0.57（0.49，0.67）	0.48（0.41，0.56）	0.37（0.32，0.44）	<0.001
Excluded lycopene	1.00	0.66（0.56，0.77）	0.64（0.55，0.75）	0.47（0.40，0.56）	<0.001
Excluded lutein and zeaxanthin	1.00	0.63（0.54，0.74）	0.50（0.42，0.59）	0.31（0.25，0.38）	<0.001
Excluded flavonoids	1.00	0.72（0.61，0.84）	0.68（0.58，0.81）	0.45（0.37，0.54）	<0.001
Excluded glucosinolates	1.00	0.54（0.46，0.63）	0.40（0.33，0.47）	0.22（0.18，0.27）	<0.001
Excluded vitamin C	1.00	0.64（0.55，0.76）	0.48（0.40，0.58）	0.23（0.18，0.28）	<0.001
Excluded vitamin E	1.00	0.71（0.60，0.82）	0.64（0.54，0.76）	0.43（0.36，0.52）	<0.001
Excluded selenium	1.00	0.64（0.55，0.75）	0.56（0.47，0.65）	0.41（0.35，0.49）	<0.001

Excluded iron	1.00	0.63（0.53，0.74）	0.50（0.42，0.61）	0.25（0.20，0.31）	<0.001
Excluded alcohol consumption	1.00	0.60（0.51，0.70）	0.55（0.46，0.64）	0.44（0.38，0.53）	<0.001
Excluded smoking history	1.00	0.78（0.67，0.91）	0.59（0.50，0.69）	0.45（0.38，0.54）	<0.001
Excluded obesity	1.00	0.71（0.61，0.83）	0.61（0.52，0.72）	0.34（0.29，0.41）	<0.001
Excluded physical activity	1.00	0.55（0.47，0.64）	0.53（0.45，0.62）	0.42（0.35，0.49）	<0.001
OBS-3					
Excluded total fat	1.00	0.79（0.68，0.93）	0.62（0.52，0.72）	0.52（0.44，0.62）	<0.001
Excluded carotene	1.00	0.80（0.69，0.94）	0.62（0.51，0.74）	0.52（0.42，0.64）	<0.001
Excluded vitamin C	1.00	0.68（0.58，0.80）	0.52（0.43，0.62）	0.40（0.32，0.49）	<0.001
Excluded vitamin E	1.00	0.77（0.65，0.90）	0.64（0.53，0.77）	0.57（0.46，0.70）	<0.001
Excluded riboflavin	1.00	0.79（0.67，0.93）	0.52（0.43，0.64）	0.60（0.48，0.75）	<0.001
Excluded niacin	1.00	0.59（0.50，0.70）	0.46（0.38，0.54）	0.31（0.25，0.37）	<0.001
Excluded vitamin B_6	1.00	0.72（0.61，0.86）	0.56（0.46，0.69）	0.51（0.40，0.66）	<0.001
Excluded total folate	1.00	0.80（0.67，0.94）	0.65（0.53，0.79）	0.67（0.53，0.84）	<0.001
Excluded vitamin B_{12}	1.00	0.79（0.68，0.92）	0.54（0.46，0.64）	0.47（0.39，0.56）	<0.001
Excluded selenium	1.00	0.77（0.66，0.90）	0.62（0.52，0.73）	0.56（0.47，0.68）	<0.001
Excluded iron	1.00	0.54（0.45，0.64）	0.33（0.26，0.41）	0.21（0.16，0.28）	<0.001
Excluded zinc	1.00	0.60（0.51，0.71）	0.33（0.28，0.40）	0.24（0.19，0.30）	<0.001
Excluded copper	1.00	0.80（0.68，0.94）	0.71（0.60，0.84）	0.55（0.45，0.67）	<0.001
Excluded calcium	1.00	0.76（0.64，0.89）	0.61（0.50，0.74）	0.69（0.55，0.85）	<0.001
Excluded magnesium	1.00	0.66（0.55，0.78）	0.43（0.34，0.53）	0.37（0.28，0.48）	<0.001
Excluded dietary fiber	1.00	0.87（0.74，1.03）	0.78（0.64，0.94）	0.71（0.57，0.89）	<0.001
Excluded alcohol consumption	1.00	0.70（0.60，0.82）	0.60（0.51，0.71）	0.50（0.42，0.59）	<0.001
Excluded smoking history	1.00	0.85（0.73，0.99）	0.65（0.55，0.76）	0.51（0.43，0.60）	<0.001
Excluded obesity	1.00	0.68（0.58，0.79）	0.56（0.48，0.66）	0.46（0.39，0.55）	<0.001
Excluded physical activity	1.00	0.87（0.75，1.02）	0.65（0.56，0.77）	0.54（0.45，0.64）	<0.001
OBS-4					
Excluded SFAs	1.00	0.74（0.63，0.86）	0.64（0.55，0.76）	0.50（0.42，0.60）	<0.001
Excluded n-6 PUFAs	1.00	0.67（0.57，0.78）	0.59（0.51，0.70）	0.43（0.36，0.51）	<0.001
Excluded n-3 PUFAs	1.00	0.81（0.69，0.94）	0.64（0.55，0.76）	0.45（0.38，0.53）	<0.001
Excluded total carotenoids	1.00	0.81（0.69，0.94）	0.51（0.43，0.61）	0.56（0.46，0.67）	<0.001
Excluded flavonoids	1.00	0.79（0.67，0.92）	0.83（0.70，0.98）	0.44（0.36，0.53）	<0.001
Excluded glucosinolates	1.00	0.59（0.51，0.70）	0.41（0.34，0.49）	0.27（0.22，0.33）	<0.001
Excluded vitamin C	1.00	0.78（0.66，0.92）	0.48（0.40，0.58）	0.48（0.39，0.59）	<0.001
Excluded vitamin E	1.00	1.00（0.86，1.17）	0.88（0.75，1.04）	0.52（0.43，0.62）	<0.001
Excluded selenium	1.00	0.89（0.76，1.03）	0.74（0.63，0.87）	0.50（0.42，0.59）	<0.001
Excluded zinc	1.00	0.57（0.48，0.66）	0.51（0.43，0.60）	0.26（0.22，0.32）	<0.001
Excluded iron	1.00	0.64（0.54，0.76）	0.42（0.34，0.51）	0.36（0.29，0.45）	<0.001
Excluded alcohol consumption	1.00	0.87（0.74，1.02）	0.72（0.61，0.84）	0.51（0.43，0.61）	<0.001
Excluded smoking history	1.00	0.85（0.72，0.99）	0.72（0.62，0.85）	0.46（0.39，0.55）	<0.001
Excluded obesity	1.00	0.70（0.60，0.82）	0.56（0.48，0.66）	0.50（0.42，0.59）	<0.001
Excluded physical activity	1.00	0.77（0.66，0.90）	0.66（0.57，0.78）	0.45（0.38，0.53）	<0.001

Abbreviations: OR, odds ratio; CI, confidence interval; Q, quartile; OBS, oxidative balance score; PUFAs, polyunsaturated fatty acids; SFAs, saturated fatty acids; BMI, body mass index. [a] OBS-1 adjusted for sex, age, marital status, residence, educational level, occupation, income, first-degree relative with cancer, total energy intake, occupational activity, household and leisure-time activities, and BMI. OBS-2, OBS-3 and OBS-4 adjusted for sex, age, marital status, residence, educational level, occupation, income, first-degree relative with cancer, total energy intake, and occupational activity.
